# Late‐Onset *Salmonella* Abscess of a Silicone Breast Implant Presenting 40 Years After Augmentation

**DOI:** 10.1155/crdi/7398033

**Published:** 2026-08-17

**Authors:** Bennett Schackmuth, Paula Santo, Jonathan Livingston, Jacob Nichols

**Affiliations:** ^1^ School of Medicine, Texas Tech University Health Sciences Center, Lubbock 79430, Texas, USA, ttuhsc.edu

**Keywords:** breast implant infection, mastitis, *Salmonella*

## Abstract

We present a rare case of breast implant infection due to *Salmonella* species in a 74‐year‐old woman who underwent breast augmentation in 1985. Forty years later, in 2025, she developed sudden left breast edema, pain, and fever. She denies any previous gastrointestinal symptoms. She was prescribed oral antibiotics without any improvement, and she was subsequently admitted to the hospital and was started on broad‐spectrum intravenous antibiotics. CT scan of the chest revealed dermal thickening of the left breast consistent with cellulitis, a fluid collection concerning for a developing abscess, and a possible implant rupture. She then underwent incision and drainage of the left breast with implant removal. Intraoperative cultures grew 4+ *Salmonella* species*.* She was switched to oral levofloxacin for a total of 2 weeks and discharged home with wound care, with subsequent clinical improvement. In our patient, the source of the *Salmonella* infection is not clear, and it was an unexpected finding. This underscores the important role of obtaining intraoperative cultures in all cases of breast implant infection.

## 1. Introduction

Breast implant infections are a known, yet relatively uncommon, complication of augmentation mammoplasty. They occur in 2%–53% of cases and are more prevalent after reconstructive surgery following breast cancer treatment than after esthetic breast augmentation [1].

Common pathogens are *Staphylococcus aureus* and coagulase‐negative staphylococci. Atypical mycobacteria and Gram‐negative bacilli have been reported but rarely [2]. Infections caused by atypical organisms such as *Salmonella* are exceedingly rare, with only two previously reported cases in the literature [3, 4].

## 2. Case Presentation

A 74‐year‐old female with a past medical history of Type 2 diabetes mellitus, hypertension, and hypothyroidism presented to the emergency department with a ten‐day history of worsening left breast pain, swelling, and erythema. Her symptoms began after a sudden jerking motion while walking her dog, which caused a “popping” sensation in her left breast. She had undergone bilateral breast augmentation with silicone implants in 1985. Two days prior to presentation, she was diagnosed with mastitis by her primary care physician and started on amoxicillin–clavulanate without improvement. She reported subjective fevers and myalgias, which had resolved, but she denied any recent gastrointestinal symptoms, including nausea, vomiting, or diarrhea. She denied any recent travel.

**FIGURE 1:**
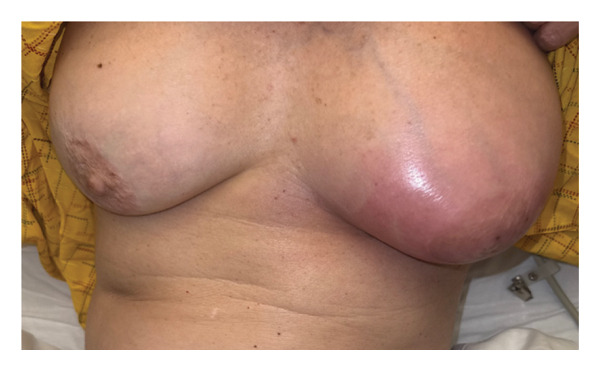
Clinical appearance of the breast showing asymmetric edema and erythema.

**FIGURE 2:**
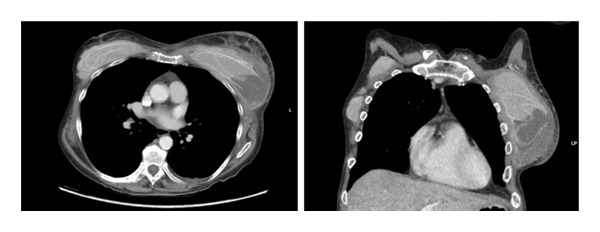
Computed tomography of the chest showing cellulitis, fluid collection, and possible implant rupture.

On physical examination, her temperature was 100.1°F (37.8°C). The left breast was erythematous, edematous, and tender to palpation, particularly on the lateral and inferior aspects. Laboratory results were significant for a white blood cell count of 18.29 K/µL. A CT scan of the chest revealed dermal thickening of the left breast consistent with cellulitis, a fluid collection concerning for a developing abscess, and a possible implant rupture. Images of the swelling and erythema are shown in Figure 1, while an axial cut of the CT scan is shown in Figure 2.

The patient was admitted and started on empiric intravenous antibiotics—vancomycin and piperacillin–tazobactam. On Hospital Day 2, she underwent incision and drainage of the left breast with implant removal. Intraoperative findings included purulent fluid and a gelatin‐like substance consistent with a ruptured silicone implant. After thorough evacuation of the wound, the middle of the wound was packed, and the skin was loosely reapproximated and sutured and covered in a sterile dressing. Tissue and fluid cultures were sent for analysis.

Postoperatively, the patient’s wound was managed with a vacuum‐assisted closure device. On Postoperative Day 2, the intraoperative cultures grew 4+ *Salmonella* species. The patient’s antibiotics were narrowed to ceftriaxone based on sensitivities. It was discovered that she frequently receives unwashed eggs from her neighbor, which may be one source of contamination. Infectious disease specialists were consulted, and her antibiotic regimen was switched to oral levofloxacin for a total of 2 weeks. The patient’s clinical condition improved, and she was discharged home on Hospital Day 9 with a plan for continued outpatient wound care. She was seen in the clinic after finishing the antibiotic course with complete resolution of her symptoms. A summary of the clinical course from presentation through outpatient follow‐up is provided in Table 1.

**TABLE 1:** Clinical timeline from presentation to follow‐up.

Day	Event
Day−10	Onset of left breast pain, swelling, and erythema following a sudden jerking motion (“popping” sensation) while walking the dog.
Day−2	Diagnosed clinically with mastitis by primary care physician; started on oral amoxicillin–clavulanate without improvement.
Day 0 (Hospital Day 1)	ED presentation: *T* 100.1°F, WBC 18.29 K/µL. CT chest: dermal thickening of left breast, fluid collection concerning for abscess, possible implant rupture. Admitted; started on IV vancomycin and piperacillin–tazobactam.
Hospital Day 2	Incision and drainage of the left breast with implant removal. Intraoperative findings: purulent fluid and gelatin‐like material consistent with ruptured silicone implant. Tissue and fluid cultures sent.
Hospital Day 4	Intraoperative cultures grew 4+ *Salmonella* species. Antibiotics narrowed to ceftriaxone per sensitivities. Infectious disease consulted; transitioned to oral levofloxacin.
Hospital Day 9	Discharged home with vacuum‐assisted wound closure and oral levofloxacin to complete a 2‐week course.
Outpatient follow‐up	Complete resolution of symptoms after antibiotic course; wound healed.

## 3. Discussion

This case is, to the best of our knowledge, the third case published in the literature of a breast implant infection due to *Salmonella* species*.* In 1995, Asaadi et al. published a case of a 39‐year‐old woman who underwent breast reconstruction with insertion of a saline implant who later developed an infection by *Salmonella choleraesuis* [3]. Another case, published in 2018 by Hall et al., involved a 34‐year‐old female who developed a *Salmonella* serogroup C breast implant infection following an episode of traveler’s diarrhea [4]. In that case, the proposed mechanism of infection was hematogenous seeding from the gastrointestinal tract.

In our patient, the source of the *Salmonella* infection is less clear. She denied any recent travel or gastrointestinal symptoms, making hematogenous seeding from a gastrointestinal source less likely, though not impossible, as asymptomatic bacteremia can occur. Her history of consuming unwashed eggs is a potential risk factor for *Salmonella* infection. It is also conceivable that she had a subclinical gastrointestinal infection that led to transient bacteremia and subsequent seeding of the breast implant. The trauma from the dog walking incident may have caused a disruption of the implant capsule, creating a favorable environment for bacterial growth. In cases of delayed unilateral breast swelling associated with peri‐implant fluid collection, a careful differential diagnosis is essential, including consideration of breast implant–associated anaplastic large cell lymphoma (BIA‐ALCL) [5].

The novelty of this case is threefold: To our knowledge, it represents the third reported *Salmonella* breast implant infection, the longest reported interval (40 years) between augmentation and infection, and the first such case occurring in the absence of preceding gastrointestinal symptoms or recent travel. With respect to laboratory data, the patient’s blood cultures were negative at 3 days; intraoperative tissue and fluid cultures grew 4+ *Salmonella* species. Despite the absence of overt enteric symptoms, hematogenous seeding from transient, subclinical gastrointestinal colonization remains the most plausible route of transmission. The patient’s regular consumption of unwashed eggs from a neighbor provides a credible exposure source, and asymptomatic *Salmonella* bacteremia from the gut is well described, particularly in older adults with comorbidities such as Type 2 diabetes mellitus. We propose a sequence in which low‐grade bacteremia from gut colonization seeded the implant capsule, with mechanical disruption from the dog‐walking incident either creating or exposing a niche on the silicone surface that favored biofilm formation and progression to clinical infection. Direct hematogenous spread from a remote infected focus and contiguous spread from skin flora are considered less likely given the absence of an identifiable distant focus and the Gram‐negative microbiology.

Mastitis caused by *Salmonella* species, but not related to breast implants, is also a rare condition, although some case reports have been published, including breast abscesses related to this pathogen [6–9]. Most cases are associated with immunocompromising conditions and/or recent travel, and hematogenous spread is the most commonly proposed cause.

The diagnosis of a *Salmonella* breast implant infection was an unexpected finding. This case is an example of the important role of intraoperative cultures in all cases of breast implant infection, as the causative organism may be unusual and not covered by empiric antibiotic therapy. The successful management of this patient involved a multidisciplinary approach with reconstructive surgery and infectious disease specialists. The patient’s treatment was based on three main components: the removal of the implant, surgical debridement, and a course of antibiotics guided by culture results.

## 4. Conclusion

Late‐onset breast implant infection caused by *Salmonella* species is exceedingly rare and may occur decades after augmentation, even in the absence of overt gastrointestinal symptoms or recent travel. Clinicians evaluating delayed peri‐implant fluid collections should maintain a broad differential that includes both atypical infectious organisms and BIA‐ALCL. This case underscores the value of obtaining intraoperative tissue and fluid cultures in all suspected breast implant infections, as empiric therapy may not cover unusual Gram‐negative pathogens, and successful management typically requires a combined approach of explanation, surgical debridement, and culture‐directed antibiotic therapy.

## Author Contributions

Bennett Schackmuth: case identification, clinical care, and manuscript drafting. Paula Santo: corresponding author, clinical care, and manuscript review. Jonathan Livingston: clinical care and manuscript review. Jacob Nichols: clinical care, supervision, and critical revision.

## Funding

No funding was received.

## Disclosure

All authors read and approved the final manuscript.

## Ethics Statement

This single‐patient case report did not constitute human subjects research per the Texas Tech University Health Sciences Center Institutional Review Board, and formal IRB review was not required. Written informed consent for publication was obtained from the patient.

## Consent

Written informed consent was obtained from the patient for the publication of this case report.

## Conflicts of Interest

The authors declare no conflicts of interest.

## Data Availability

All data relevant to this case report are included within the manuscript. Additional deidentified data are available from the corresponding author upon reasonable request.
